# LSD1 modulates the bone metastasis of breast cancer cells through hnRNPA2B1-mediated sorting of exosomal miRNAs

**DOI:** 10.1038/s41420-024-01897-5

**Published:** 2024-03-06

**Authors:** Ziyu Liu, Benkai Xin, Nan Zhang, Peipei An, Yueru Shi, Jingtong Yang, Youzhong Wan, Yuquan He, Xin Hu

**Affiliations:** 1 China-Japan Union Hospital of Jilin University, Jilin University, Changchun, Jilin 130033 China; 2 School of Life Sciences, Jilin University, Changchun, Jilin 130012 China

**Keywords:** Cancer microenvironment, Breast cancer

## Abstract

Bone metastasis is a key contributor to morbidity and mortality of breast cancer patients. We have previously shown that exosomal miRNAs derived from LSD1 knockdown (KD) breast cancer cells inhibit osteoblast differentiation and promote osteoclast differentiation. However, how LSD1 regulates exosomal miRNAs and whether miRNAs promote bone metastasis through the formation of pre-metastatic niches remains unclear. In vivo experiments demonstrates that exosomes derived from LSD1 KD breast cancer cells significantly promoted bone metastasis. To explore the mechanism underlying the effect of LSD1 on exosomes in breast cancer cells, exosomal and cellular miRNAs from control, LSD1 KD, and rescue cells were sequenced. Interestingly, approximately 80% of LSD1-associated miRNAs were downregulated in exosomes from LSD1 KD cells. The consensus sequence UAGGGC, was identified in many miRNAs downregulated in LSD1 KD exosomes. We found that hnRNPA2B1 regulated the exosomal sorting of miR-6881-3p and some other miRNAs. LSD1 deficiency reduced hnRNPA2B1 expression in breast cancer cells by decreasing the level of H3K9me2 demethylation in the promoter region of the hnRNPA2B1 gene. Our study revealed that LSD1 plays a crucial role in the regulation of exosomal sorting of miRNA.

## Introduction

Metastasis accounts for approximately 90% of cancer-related deaths, while bone is one of the most common targets of metastatic cells [1, 2]. The growth of metastatic lesions disrupts tissue homeostasis, thereby promoting cancer cell survival and proliferation [3]. Skeletal-related events among patients with bone metastasis include hypercalcemia, osteolysis, bone fractures, spinal cord compression, and bone marrow aplasia, which severely impact the quality of life of affected individuals. Furthermore, patients with bone metastasis have poor life expectancy and prognosis. Breast cancer is the leading cause of cancer-related death in women, and bone metastasis from breast cancer is incurable.

Exosomes are cell-secreted extracellular membrane vesicles secreted by cells and typically range from 30 to 150 nm in diameter [4]. They mediate intercellular communication through encapsulated biomolecules such as proteins, lipids, RNA, and metabolites [5]. Recent evidence suggests that breast cancer-derived exosomal miRNAs contribute to bone metastasis. For instance, it was reported that MDA-MB-231 breast cancer cell-derived exosomal miR-20a-5p inhibits the expression of SRC kinase signal inhibitor 1 (SRCIN1) and promotes osteoclast formation [6]. Additionally, miR-21 derived from MDA-MB-231 and SCP28 exosomes was found to be highly expressed in serum exosomes of breast cancer patients with bone metastasis, which altered the protein level of programmed cell death 4 (PDCD4), thus contributing to the generation of pre-metastatic niches, the promotion of osteoclast differentiation, and the enhancement of bone metastasis [7]. Furthermore, exosomes obtained from estrogen receptor (ER)-positive breast cancer cells were observed to transfer miR-19a to osteoclast precursors, which promoted osteoclast differentiation by reducing the expression of phosphatase and tensin homolog (PTEN), thereby activating the nuclear factor-kappa B (NF-κB) and protein kinase B (AKT) signaling pathways [8].

Evidence increasingly supports that miRNAs are not randomly exported to exosomes; instead, specific exo-motifs in miRNAs may be recognized by RNA binding proteins, leading to miRNA sorting into exosomes [9]. HnRNPA2B1 is an RNA-binding protein capable of recruiting miRNAs into exosomes. For example, Villarroya-Beltri et al. [10] identified GGAG/UGCA as being exo-motifs recognized by hnRNPA2B1 in miR-198 and miR-601 that regulate their loading into exosomes. Meanwhile, Wu et al. showed that hnRNPA2B1 contains two RNA recognition motifs (RRMs) that can bind to AGG and UAG motifs in RNA in a sequence-specific manner and determined the crystal structure of hnRNPA2B1 complexed with several RNA substrate. Additionally, Lee et al. [11] demonstrated that hnRNPA2B1 traffics miR-17 and miR-93 to macrophages by recognizing AGG and UAG motifs. Other RNA binding proteins, such as Y-box binding protein 1 (YBX1) [12], La [13], and the translational regulator fragile X messenger ribonucleoprotein 1 (FMRP1) [14], can also recognize and traffic miRNAs into exosomes *via* conserved motifs. Garcia-Martin et al. [9] explored the sorting sequences of exosomal miRNAs released from five different cell types and found that miRNA sequences differed between miRNAs sorted to exosomes and those retained in cells. Additionally, miRNAs in exosomes released from different cells were found to contain cell type-specific conservative motifs. By analyzing the most conserved motifs, such as CGGGAG, RNA binding proteins Aly/REF export factor (ALYREF) and fused in sarcoma (FUS) were identified to promote miRNA enrichment in exosomes *via* these motifs [9].

Lysine-specific demethylase 1 (LSD1) is a histone demethylase [15] that selectively demethylates histone H3 lysine 4 (H3K4) and represses gene expression in a flavin adenine dinucleotide (FAD)-dependent manner [15]. In contrast to its interaction with H3K4, which suppresses transcription, when LSD1 associates with the androgen receptor, it acts as an oncogene *via* the mono- and di-methylation of histone H3 lysine 9 (H3K9), resulting in the activation of transcription of a variety of genes [16]. As a component of the nucleosome-remodeling and deacetylase (NuRD) complex, LSD1 inhibits the metastasis of MDA-MB-231 cell-derived tumors in immunodeficient mice by regulating the transforming growth factor beta (TGF-β) signaling pathway [17]. The LSD1/NuRD complex can bind to super-enhancers by interacting with BRD4, thus conferring widespread drug resistance in breast cancer [18]. LSD1 also suppresses the proliferative and invasive ability of breast cancer cells by interacting with ZNF516-CtBP/CoREST and inhibits breast cancer growth and metastasis in vivo [19]. In addition, LSD1 suppresses breast cancer stem cell (CSC) properties by inhibiting epithelial–mesenchymal transition through its interaction with ubiquitously transcribed tetratricopeptide (UTX) and histone deacetylase 1 (HDAC1) [20]. We have shown that LSD1 suppresses breast cancer metastasis by inhibiting the expression of tripartite motif containing 37 (TRIM37) and inducing the transcription of GATA binding protein 3 (GATA3) [21].

In our previous study, we reported that the knockdown of LSD1 in breast cancer cells resulted in the downregulation of miR-6881-3p in exosomes, which inhibited the differentiation of osteoblasts and promoted osteoclasts through modulation of the expression of the target genes pre-B-cell leukemia transcription factor 1 (*PBX1*) and additional sex combs-like 2 (*ASXL2*) respectively [22]. In this study, we sought to determine whether exosomes derived from LSD1 KD breast cancer cells could promote breast cancer metastasis to bone by remodeling the bone microenvironment, and explored the molecular mechanism underlying the LSD1-modulated miRNA expression in exosomes. Our findings showed that LSD1 plays a crucial role in remodeling the pre-metastatic niche of breast cancer bone metastasis by regulating exosomal miRNA levels.

## Results

### Exosomes from LSD1 KD breast cancer cells promoted bone metastasis

**Fig. 1:**
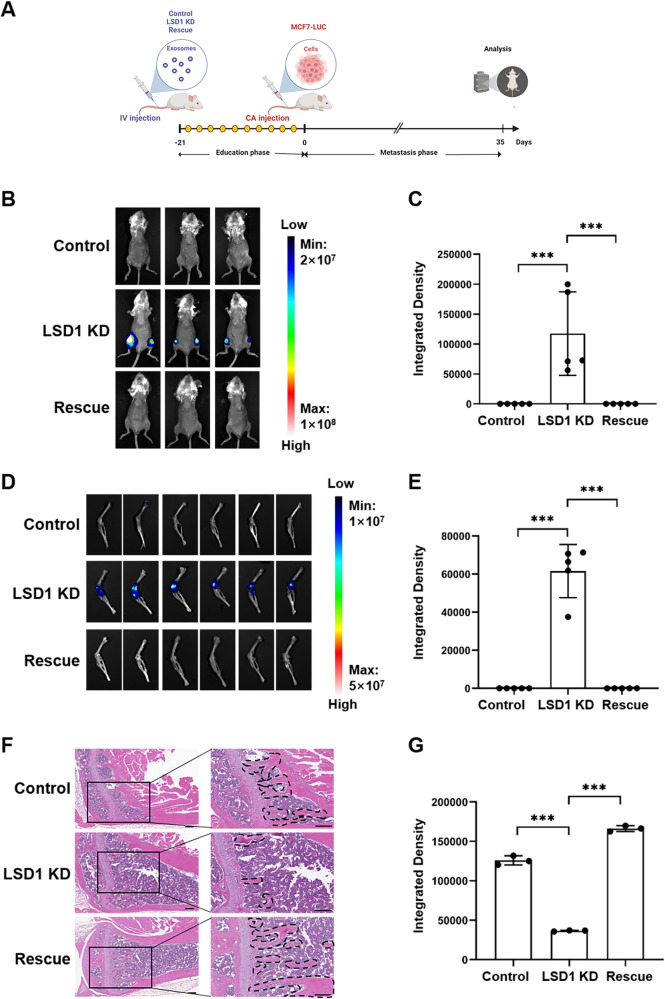
Exosomes from LSD1 knockdown (KD) breast cancer cells promoted bone metastasis. **A** Schedule of animal experiments. **B** Representative images of in vivo imaging of mice treated with exosomes from control MCF7 cells, LSD1 KD MCF7 cells, or LSD1 KD MCF7 cells expressing the LSD1 Rescue plasmid (Rescue). **C** Quantitative analysis of luminescence in mice (*n* = 5 per group). **D** Representative images of luminescence imaging of the femur and tibia of mice. **E** Quantitative analysis of luminescence of the femur and tibia of mice (*n* = 5). **F** Images of hematoxylin and eosin (H&E) staining of the proximal tibia derived from mice treated with exosomes as indicated (scale bars: 100 μm). **G** Quantitative analysis of the bone area in the tibia (*n* = 3 per group). *** indicates *P* < 0.001.

Exosomes were isolated from control, LSD1 KD, and LSD1 Rescue MCF7 cells, and exosomal markers CD63 and TSG101 were enriched in the isolated exosomes, which showed cup-shaped bilayer membranes (Fig. S1A, B). In addition, NTA analysis showed that the sizes of the exosomes were between 140 and 150 nm (Fig. S1C). Meanwhile, PKH67 labeled exosomes were cultured with hBMSCs and RAW264.7 cells, and fluorescence staining showed that these exosomes could be absorbed by hBMSCs and RAW264.7 cells (Fig. S1D). Then we analyzed the distribution of exosomes in vivo at 24 h post intravenous injection, which demonstrated that the exosomes exhibited the propensity to reach the bone (Fig. S2A, B). Exosomes from control, LSD1 KD, and LSD1 Rescue MCF7 cells were injected into mice once every other day for a total of 10 times, and then MCF7-Fluc cells stably expressing firefly luciferase were injected through the tail artery (Fig. 1A). After 5 weeks, in vivo imaging analysis was performed on each group of mice, which demonstrated that the fluorescence intensity was significantly increased in the posterior limb of mice treated with LSD1 KD exosomes compared with that in the posterior limb of mice treated with control and LSD1 Rescue exosomes (Fig. 1B, C). Specifically, bioluminescence intensity in the femur and tibia of mice treated with LSD1 KD exosomes was upregulated compared with that in the other two groups (Fig. 1D, E). Consistent with these observations, H&E staining results showed that the bone trabecula was damaged and sclerotin levels were reduced in mice treated with LSD1 KD exosome (Fig. 1F, G). These results showed that exosomes derived from LSD1 KD breast cancer cells significantly promoted osteolytic metastasis of breast cancer cells.

### Exosomes released from LSD1 KD breast cancer cells induced osteolysis

**Fig. 2:**
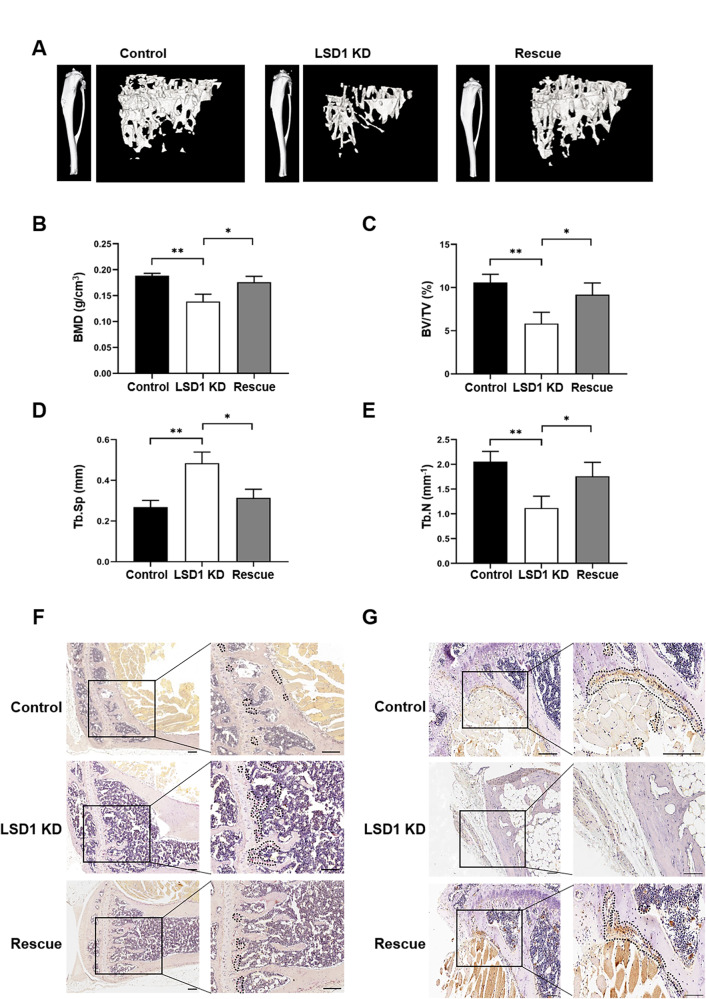
Exosomes from LSD1 knockdown (KD) breast cancer cells induced osteolysis. **A** Micro-computed tomography (micro-CT) 3D reconstruction of the cortical and trabecular structures of mice treated with exosomes from control, LSD1 KD, and LSD1 Rescue breast cancer cells. The statistical analysis of bone mineral density (BMD) (**B**), trabecular bone volume (BV/TV) (**C**), trabecular separation (Tb.Sp) (**D**), and trabecular number (Tb.N) (**E**) of the proximal tibia from mice treated with exosomes as indicated (*n* = 3). **F** Representative images of TRAP staining of the mouse tibia in the control, LSD1 KD, and LSD1 Rescue exosomal groups (scale bars: 100 μm). **G** Representative images of immunohistochemical staining for OCN in the tibia of mice (scale bars: 100 μm). * indicates *P* < 0.05, ** indicates *P* < 0.01.

To further investigate the effect of exosomes released from LSD1 KD breast cancer cells on the structure of bone, the tibias of mice were scanned by micro-CT. Three-dimensional reconstruction of the obtained images demonstrated that the bone trabeculae in the tibia of mice in the LSD1 KD group had experienced significant damage (Fig. 2A). BMD, BV/TV, Tb.N, and Tb.Sp were calculated based on micro-CT results. We found that BMD, BV/TV, and Tb.N were reduced, whereas Tb.Sp was significantly increased in the LSD1 KD group, demonstrating that LSD1 KD exosomes promoted breast cancer bone metastasis and led to bone loss (Fig. 2B–E). Additionally, there were more TRAP-stained cells in the tibia of LSD1 KD mice compared with that in control and LSD1 Rescue animals (Fig. 2F). In contrast, immunohistochemical staining for OCN was reduced in the LSD1 KD mouse tibia (Fig. 2G). Together, these results suggested that exosomes derived from LSD1 KD breast cancer cells promoted the bone metastasis of breast cancer cells by destroying tibial trabeculae and induced osteolysis by increasing the number of osteoclasts and decreasing that of osteoblasts.

### The knockdown of LSD1 decreased the levels of some miRNAs in exosomes

**Fig. 3:**
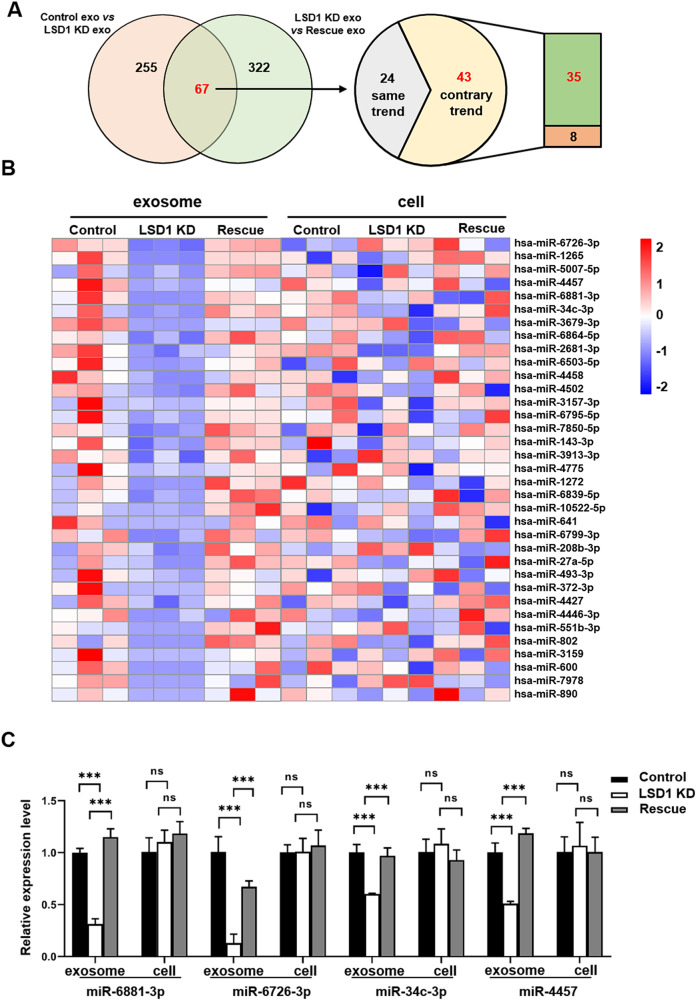
The knockdown (KD) of LSD1 decreased the levels of some miRNAs in exosomes. **A** Venn diagrams of the numbers of miRNAs that were differentially expressed in control, LSD1 KD, and LSD1 Rescue exosomes. **B** Cluster heatmap of exosomal or cellular miRNA in the control, LSD1 KD, and LSD1 Rescue groups. **C** Relative expression levels of miR-6881-3p, miR-6726-3p, miR-34c-3p, and miR-4457 from control, LSD1 KD, and LSD1 Rescue cells and exosomes as determined by qPCR assay. *** indicates *P* < 0.001.

In our previous study, we conducted a miR-seq analysis and found that the LSD1 KD inhibited the differentiation of osteoblasts and promoted that of osteoclasts by downregulating exosomal miR-6881-3p levels [22], presumably leading to osteolytic bone metastasis. Here, we further explored the specific molecular mechanism underlying these effects of LSD1 KD. Re-analysis of the exosomal miR-seq data indicated that the levels of 81.4% (35/43) of LSD1-related miRNAs were decreased in LSD1 KD exosomes, and expression of these 35 miRNAs was reversed in exosomes from LSD1 Rescue cells (Fig. 3A). Accordingly, we also performed miR-seq on cells in this study and analyzed the expression of these miRNAs in both cells and exosomes from the control, LSD1 KD, and LSD1 Rescue groups. Interestingly, the expression of these 35 miRNAs did not differ significantly among the cells of the three groups, but was downregulated in LSD1 KD exosomes relative to that in control and LSD1 Rescue exosomes (Fig. 3B). Real-time PCR assays confirmed that the expression of miR-6881-3p, miR-6726-3p, miR-34c-3p, and miR-4457 was decreased in LSD1 KD exosomes and increased in LSD1 Rescue exosomes. Meanwhile, no difference in the expression of these miRNAs was observed among cells of the respective groups (Fig. 3C). These observations showed that LSD1 KD resulted in the downregulation of 81.4% of LSD1-related miRNAs in exosomes, whereas the expression of most of these miRNAs did not differ among the respective cells. These findings suggested that LSD1 did not affect the expression of these miRNAs but likely influenced their sorting into exosomes.

### HnRNPA2B1 bound to the downregulated miRNAs in LSD1 KD exosomes

**Fig. 4:**
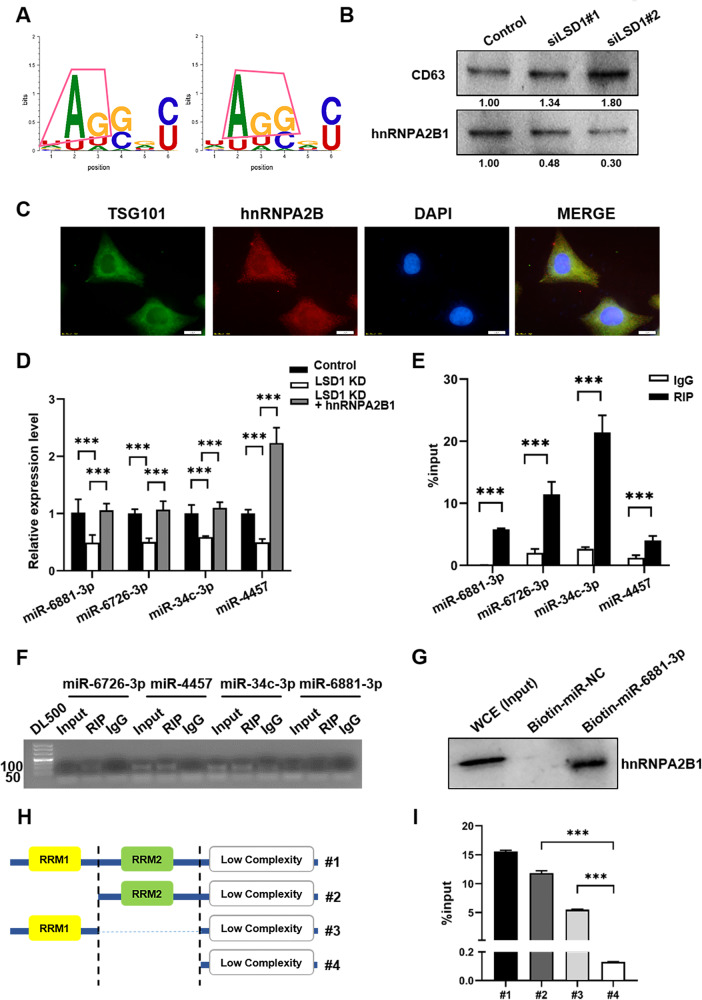
HnRNPA2B1 bound to the downregulated miRNAs in LSD1 knockdown (KD) exosomes. **A** Diagram of the over-represented motifs found in the downregulated miRNAs in LSD1 KO exosomes. **B** Western blots showing the expression of CD63 and hnRNPA2B1 in exosomes purified from control and LSD1 KD cells. The numbers below the bands represent the value from densitometry reading by Image J software, and the Control was set at 1.00 arbitrarily. **C** Representative images of TSG101 and hnRNPA2B1 co-localization in MCF7 cells as determined by immunofluorescence assay (green: hnRNPA2B1; red: TSG101; blue: DAPI; scale bars: 10 μm). **D** Relative expression levels of miR-6881-3p, miR-6726-3p, miR-34c-3p, and miR-4457 in exosomes derived from control, LSD1 KD, and LSD1 KD+hnRNPA2B1 cells. **E** Relative amounts of miR-6881-3p, miR-6726-3p, miR-34c-3p, and miR-4457 pulled down in RNA immunoprecipitation assays using hnRNPA2B1 (RIP) and control (IgG) antibodies. **F** Nucleic acid gel electrophoresis showed the amplified miR-6881-3p, miR-6726-3p, miR-34c-3p, and miR-4457. **G** Western blot showing hnRNPA2B1 pulled down by biotinylated miR-6881-3p in whole MCF7 cell extracts. **H** Structure of flag-tagged partial hnRNPA2B1. **I** Relative amounts of miR-6881-3p pulled down in RIP assay with flag-tagged full-length and truncated hnRNPA2B1. #1: full-length hnRNPA2B1 with both RRM1 and RRM2 domains; #2: truncated hnRNPA2B1 with RRM2 domain only; #3: truncated hnRNPA2B1 with RRM1 domain only; #4: truncated hnRNPA2B1 with deletion of both RRM1 and RRM2 domains. *** indicates *P* < 0.001.

An unbiased search for over-represented sequence motifs using Improbizer identified a conserved UAGGGC motif in these 35 miRNAs (Fig. 4A). HnRNPA2B1 is an RNA-binding protein that can bind to RNAs with AGG and UGA motifs [23]. To verify whether hnRNPA2B1 could bind to miRNAs in exosomes, we first determined whether hnRNPA2B1 was expressed in exosomes, and found that it was indeed present in control and LSD1 KD exosomes (Fig. 4B). Immunofluorescence assays in MCF7 cells showed that hnRNPA2B1 co-localized with TSG101, which functions as a component of the endosomal sorting complex required for transport (ESCRT) complex and participates in exosome secretion (Fig. 4C). To identify the role of LSD1 in hnRNPA2B1-mediated exosomal miRNA sorting, we transfected LSD1 KD cells with hnRNPA2B1 and found that the overexpression of hnRNPA2B1 could restore the relative levels of miR-6881-3p, miR-6726-3p, miR-34c-3p and miR-4457 in LSD1 KD exosomes, as determined by qPCR analysis (Fig. 4D). Subsequently, RIP assays confirmed that hnRNPA2B1 bound directly to these four miRNAs (Fig. 4E, F) and RNA pull-down assays confirmed that hnRNPA2B1 bound directly to miR-6881-3p (Fig. 4G). HnRNPA2B1 has two RNA-binding domains, RRM1 and RRM2. To determine which domain is involved in binding to miR-6881-3p, flag-tagged full-length and truncated hnRNPA2B1 were constructed (Fig. 4H). RIP assays demonstrated that truncated hnRNPA2B1 with RRM1 domain only and truncated hnRNPA2B1 with RRM2 domain only showed significantly higher binding to miR-6881-3p than truncated hnRNPA2B1 with deletion of both RRM1 and RRM2 domains, suggesting that both RRM1 and RRM2 domain could bind to miR-6881-3p (Fig. 4I). Furthermore, full-length hnRNPA2B1 with both RRM1 and RRM2 domains showed increased binding to miR-6881-3p as compared to truncated hnRNPA2B1 with RRM1 domain only and truncated hnRNPA2B1 with RRM2 domain only, suggesting that RRM1 and RRM2 domains of hnRNPA2B1 might have synergistic effects on the binding of miR-6881-3p (Fig. 4I). Overall, our data showed that hnRNPA2B1 could bind to some miRNAs and mediate their exosomal sorting.

### LSD1 regulated the expression of hnRNPA2B1

**Fig. 5:**
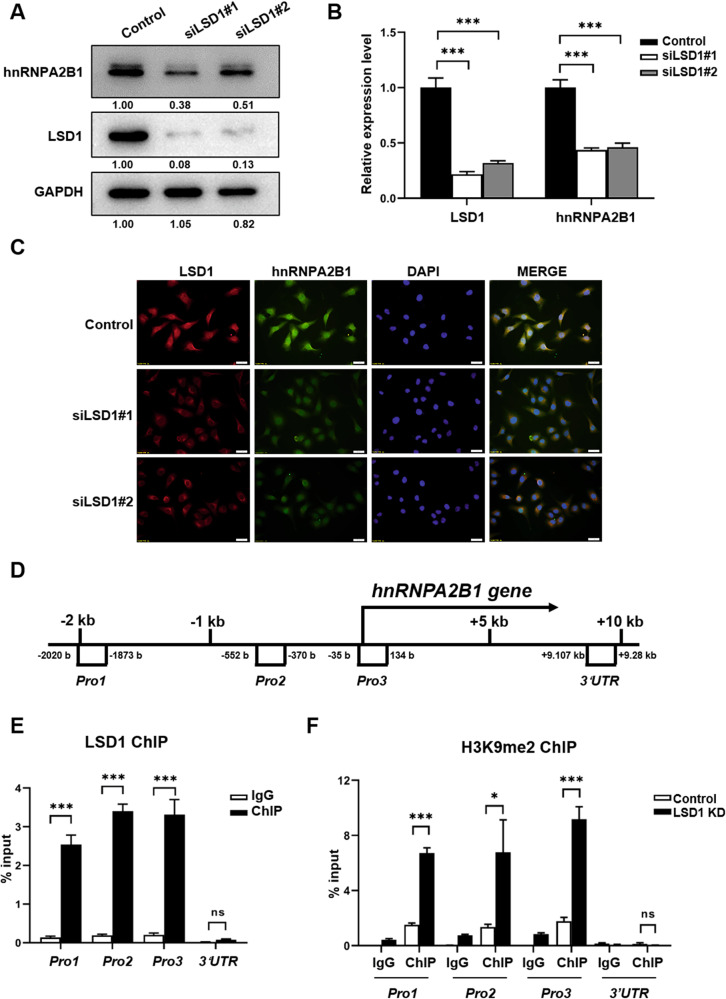
LSD1 regulated the expression of hnRNPA2B1. **A** Western blot of hnRNPA2B1 and LSD1 in control and LSD1 knockdown (KD) MCF7 cells. The numbers below the bands represent the value from densitometry reading by Image J software, and the Control was set at 1.00 arbitrarily. **B** Quantitative analysis of hnRNPA2B1 and LSD1 in control and LSD1 KD MCF7 cells by qPCR. **C** Representative images of the expression of hnRNPA2B1 and LSD1 in control and LSD1 KD MCF7 cells using immunofluorescence assays (green: LSD1; red: hnRNPA2B1; blue: DAPI; scale bars: 20 µm). **D** PCR primer sites in the hnRNPA2B1 promoter and 3′UTR used in the chromatin immunoprecipitation (ChIP) analysis. **E** Relative levels of LSD1 binding sites in the *hnRNPA2B1* promoter and 3′UTR as determined by ChIP assays. **F** Relative levels of H3K9me2 binding sites in the *hnRNPA2B1* promoter and 3′UTR regions in control and LSD1 KD MCF7 cells as determined by ChIP assay. * indicates *P* < 0.05, *** indicates *P* < 0.001.

We further investigated whether LSD1 could regulate the expression of hnRNPA2B1. Western blot and qPCR analysis showed that the protein and mRNA levels of hnRNPA2B1 were decreased in LSD1 KD cells compared with those in the controls (Fig. 5A, B). Similar results were obtained using immunofluorescence staining (Fig. 5C). ChIP assays further showed that LSD1 could bind to the promoter region of the *hnRNPA2B1* gene (Fig. 5D, E). H3K9me2 ChIP assays showed that the LSD1 KD resulted in a decrease in the methylation level of H3K9 in the *hnRNPA2B1* promoter region (Fig. 5F). In summary, LSD1 KD in breast cancer cells led to a decrease in H3K9me2 demethylation in the promoter region of *hnRNPA2B1*, resulting in its downregulation.

### HnRNPA2B1 knockdown inhibited osteoblast differentiation and promoted osteoclast differentiation by reducing the miR-6881-3p in exosomes

**Fig. 6:**
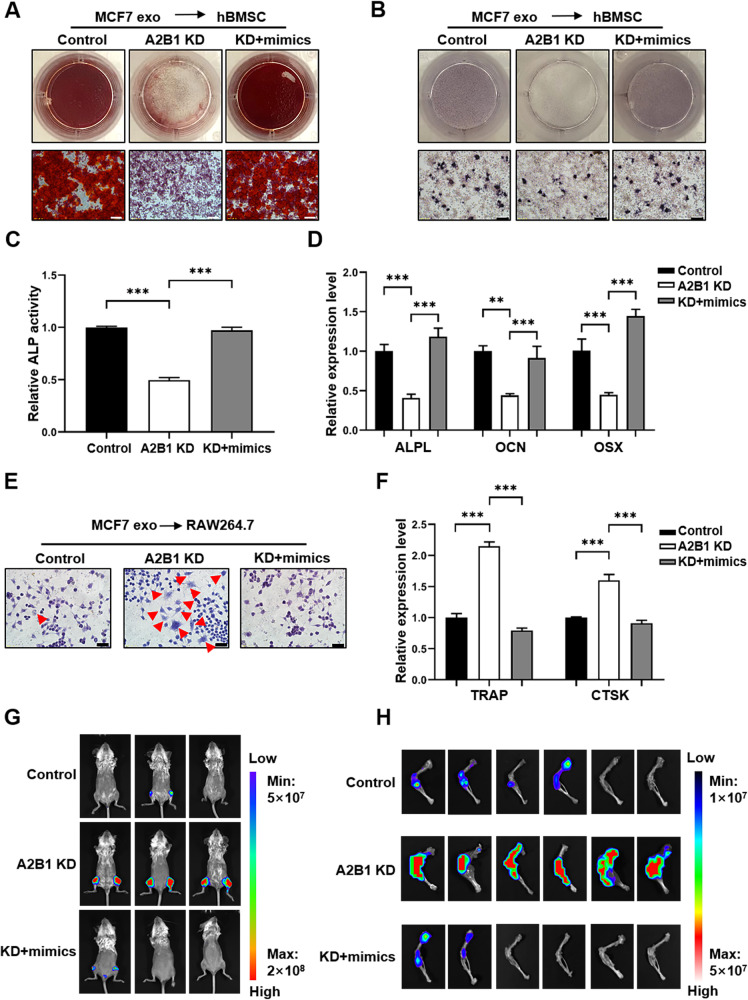
HnRNPA2B1 knockdown (KD) inhibited osteoblast differentiation and promoted osteoclast differentiation by downregulating miR-6881-3p in exosomes. Representative images of Alizarin Red staining (**A**) and ALP staining (**B**) (scale bars: 50 μm). **C** Relative ALP activity in the control, hnRNPA2B1 KD, and hnRNPA2B1KD+miR-6881-3p mimics groups. **D** qPCR analysis of the relative expression of *ALPL*, *OCN*, and *OSX* in human bone marrow mesenchymal stem cells (hBMSCs) in the control, hnRNPA2B1 KD, and hnRNPA2B1 KD+miR-6881-3p mimics groups. **E** TRAP staining in RAW264.7 cells treated with exosomes derived from control, hnRNPA2B1 KD, or hnRNPA2B1 KD+miR-6881-3p mimics-treated induced to osteoclast differentiation (scale bars: 20 µm). **F** qPCR analysis of the relative expression levels of *TRAP* and *CTSK* in RAW264.7 cells transfected with the indicated exosomes. **G** Representative images of in vivo imaging of mice treated with exosomes from control MCF7 cells, hnRNPA2B1 KD MCF7 cells, or hnRNPA2B1 KD+miR-6881-3p mimics MCF7 cells. **H** Representative images of luminescence imaging of the femur and tibia of mice. *** indicates *P* < 0.001.

We next investigated whether hnRNPA2B1 KD breast cancer cells could inhibit osteoblast differentiation and promote osteoclast differentiation by downregulating miR-6881-3p level in exosomes. Alizarin Red S staining assays showed that exosomes from hnRNPA2B1 KD breast cancer cells inhibited the formation of calcium nodules, an effect that was reversed by exosomes from hnRNPA2B1 KD breast cancer transfected with miR-6881-3p mimics (Fig. 6A & S3A, Fig. S4A, Fig. S5A). This result demonstrated that the KD of hnRNPAB21 inhibited osteoblast differentiation. ALP staining and activity analysis further demonstrated that hnRNPA2B1 KD exosomes inhibited both the production (Fig. 6B & S3B, Fig. S4B, Fig. S5B) and activity (Fig. 6C) of ALP. Moreover, qPCR assays demonstrated that exosomes from hnRNPA2B1 KD breast cancer cells inhibited the expression of the osteoblast differentiation marker genes *ALPL*, *OCN* and *OSX*, an effect that was reversed by exosomes from hnRNPA2B1 KD breast cancer cells transfected with miR-6881-3p mimics (Fig. 6D). RAW264.7 cells were used to analyze the effects of exosomes from control, hnRNPA2B1 KD and hnRNPA2B1 KD+miR-6881-3p mimics-expressing breast cancer cells on osteoclast differentiation. Interestingly, exosomes from hnRNPA2B1 KD cells promoted the differentiation of monocytes into multinuclear osteoclast-like cells, in contrast to monocytes treated by the exosomes from the control and hnRNPA2B1 KD+miR-6881-3p mimics cells (Fig. 6E & S3C, Fig. S4C, Fig. S5C). Moreover, exosomes from the hnRNPA2B1 KD cells increased the expression of both *TRAP* and *CTSK*, two well established osteoclast marker genes (Fig. 6F). The results of animal experiments also showed that the fluorescence intensity was significantly increased in the posterior limb of mice treated with hnRNPA2B1 KD exosomes compared with that in the posterior limb of mice treated with the exosomes from the control and hnRNPA2B1 KD+miR-6881-3p mimics cells (Fig. 6G). Femur and tibia scans showed that the bioluminescence intensity was upregulated in the hnRNPA2B1 KD exosomes treated group relative to that in the other two groups (Fig. 6H). Together, these results demonstrated that exosomes from hnRNPA2B1 KD breast cancer cells inhibited the differentiation of osteoblasts and promoted osteoclasts by downregulating exosomal miR-6881-3p levels, which in turn promotes bone colonization of breast cancer cells.

## Discussion

**Fig. 7:**
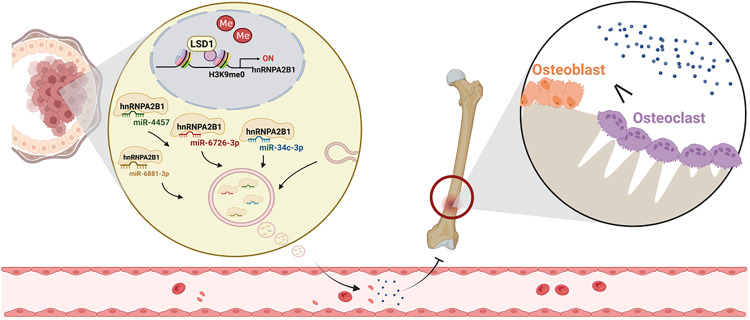
Schematic illustration of how LSD1 modulates the formation of bone pre-metastatic niches by regulating exosomal sorting. The histone demethylase LSD1 can catalyze the demethylation of histone H3K9 in the promoter region of *hnRNPA2B1*, thus regulating its transcription in breast cancer cells. HnRNPA2B1 can specifically bind to and sort miRNAs such as miR-6881-3p, miR-6726-3p, miR-34c-3p, miR-4457 into exosomes. These exosomal miRNAs further remodel the pre-metastatic niches by regulating osteoblast and osteoclast differentiation. Created with BioRender.com.

In this study, we found that exosomes from LSD1 KD breast cancer cells significantly promoted breast cancer bone metastasis. We further found that the levels of more than 80% of LSD1-related miRNAs were downregulated in LSD1 KD exosomes, but restored in exosomes derived from LSD1 KD cells expressing the LSD1 Rescue plasmid. However, the expression level of these miRNAs was not affected in the LSD1 KD cells. The consensus motif UAGGGC was found to be present in exosomal miRNAs regulated by LSD1, and this motif was recognized by hnRNPA2B1. The KD of hnRNPA2B1 reduced exosomal miR-6881-3p levels, which inhibited osteoblast differentiation and promoted osteoclast differentiation. In summary, our results demonstrated that LSD1 controls the sorting of miR-6881-3p into exosomes by regulating expression of hnRNPA2B1, which remodels the pre-metastatic niche during breast cancer bone metastasis (Fig. 7).

Exosomes play an important role in cancer metastasis. However, relatively few studies have investigated the role of exosomes in breast cancer metastasis to bone. Yuan et al. [7] found that exosomal miR-21 secreted by breast cancer cells led to the downregulation of PDCD4 in osteoclasts, thus promoting osteoclast differentiation and bone metastasis. Wu et al. [8] found that exosomal miR‐19a derived from estrogen receptor-positive breast cancer cells promoted osteoclast differentiation and bone metastasis, because miR-19a inhibits the expression of PTEN, thereby activating the NF-κB and AKT pathways in osteoclasts, which would create a microenvironment favorable for colonization in the bone [8]. Guo et al. reported that miR-20a-5p secreted by MDA-MB-231 cells enters osteoclasts *via* exosomes and promotes the proliferation and differentiation of osteoclasts by targeting SRCIN1 [6]. These studies focused on the role of exosomal miRNAs in regulating breast cancer metastasis to bone by promoting osteoclast differentiation, whereas we found that LSD1 KD can simultaneously inhibit osteoblast differentiation and promote osteoclast differentiation by downregulating miR-6881-3p, leading to the formation of pre-metastatic niche and the promotion of breast cancer bone metastasis.

An increasing number of studies have shown that the level of RNA, including miRNA, differs between exosomes and parent cells. This implies the existence of a sorting mechanism for exosomal RNA [24, 25]. In this study, we found that LSD1 KD resulted in the downregulation of approximately 80% of LSD1-related exosomal miRNAs and these miRNAs contained AGG/UAG motifs that could be recognized by hnRNPA2B1. We further found that LSD1 KD led to the downregulation of hnRNPA2B1 in breast cancer cells. However, exosomal miR-seq analysis also demonstrated that almost 20% of miRNAs were upregulated in LSD1 KD exosomes. This observation suggests that RNA binding proteins might also negatively regulate exosomal miRNA sorting, consistent with Pérez-Boza et al.’s report which found that hnRNPA2B1 inhibits the exosomal export of miR-503 in endothelial cells [26].

In addition to hnRNPA2B1, other hnRNP family members, including hnRNPA1, hnRNPC1, and hnRNPQ, have been reported to be involved in exosomal miRNA sorting. For instance, Gao et al. [27] observed that hnRNPA1 selectively sorts miR-320 into exosomes by binding to AGAGGG motifs on miR-320, while Balaguer et al. found that hnRNPC1 regulates the sorting of miR-30d into endometrial exosomes [28]. In addition, hnRNPQ, another member of the hnRNP family, was found to contain an RNA binding domain that could bind to GGCU motifs on miRNAs and sort them into exosomes [29]. These RNA-binding proteins belong to the same family as hnRNPA2B1, so they potentially have partial gene sequence similarity. In this study, we identified UAGGGC as the consensus sequence among the downregulated miRNAs in LSD1 KD exosomes. Because of the presence of AGG and UAG, these miRNAs could be sorted by hnRNPA2B1 into exosomes. However, some miRNAs that were downregulated in LSD1 KD exosomes did not contain AGG and UAG sequences. We speculate that LSD1 KD may affect the expression of other hnRNP family members, thus influencing the sorting of these miRNAs into exosomes.

Recent evidence has suggested that exosomes are involved in tumor initiation and progression. Our findings also confirmed that exosomes derived from LSD1 KD breast cancer cells promoted osteolysis, thereby remodeling the microenvironments of metastasis of bone. This suggests that targeting cancer-related exosomal miRNAs may represent a feasible strategy for inhibiting tumor metastasis. It has been reported that inhibition of exosome release can inhibit tumor proliferation, metastasis, and chemoresistance that were caused by exosomes in cancer [30]. Reserpine is an antihypertensive drug that can prevent normal cells from absorbing cancer-related exosomes and restrain the formation of pre-metastatic niches in lung metastasis [31]. Similarly, Dynasore, a GTP enzyme inhibitor, chemotherapy-resistant lymphoma patients, likely due to its ability to block the uptake of exosomes by target cells through the blocking of a cholesterol-dependent pathway [32]. Therefore, blocking the uptake of cancer-related exosomes by target cells holds promise as a strategy for inhibiting tumor occurrence and development.

## Materials and methods

### Cell culture

MCF7 and 4T1 cells were obtained from ATCC and cultured in Dulbecco’s modified Eagle’s medium (DMEM) (Gibco, NY, USA) and Roswell Park Memorial Institute (RPMI)-1640 medium (Gibco, NY, USA) supplemented with 10% fetal bovine serum (FBS) (BI, Israel) and 1% penicillin-streptomycin (Solarbio, Beijing, China). Human bone marrow mesenchymal stem cells (hBMSCs) were purchased from Cyagen Biosciences Company and cultured in OriCell Basal Medium containing Fetal Bovine Serum + Culture Supplements (Cyagen Biosciences, Santa Clara, CA, USA). RAW264.7 cells were purchased from FuHeng Biology (Shanghai, China) and cultured in DMEM containing 10% FBS and 1% penicillin-streptomycin. All the cells were incubated at 37 °C with 5% CO_2_ in a humidified incubator.

### Cell transfection

Small interfering RNAs (siRNAs) targeting LSD1 and hnRNPA2B1 were synthesized by Shanghai GenePharma Co., Ltd. The sequences of the siRNAs are listed in Table S1. The LSD1 rescue plasmid pcDNA3.1-LSD1 and the hnRNPA2B1 overexpression plasmid were synthesized by Generay Biotechnology (Shanghai, China). All the siRNAs and vectors were transfected using Lipofectamine 3000 (Invitrogen, Carlsbad, CA, USA).

### Exosome isolation

Cells were cultured first in medium supplemented with 10% FBS for 24 h and then in serum-free medium for another 36 h after transfection. The cell supernatant was collected and centrifuged to remove cells and debris and filtered through a 0.22 μm filter (Millipore, Massachusetts, USA). Then, 0.5 volumes of Total Exosome Isolation Reagent (Invitrogen, Carlsbad, USA) was added followed by incubation at 4 °C overnight. The next day, the supernatant was centrifuged at 10,000 × *g* for 1 h at 4 °C and the precipitate was collected.

### Animal experiments

This study was conducted and guided according to the Guide for the Care and Use of Laboratory Animals of the National Institutes of Health, approved by the Animal Ethics Committee of the Changchun Wish Technology Company. All mice were maintained in Changchun Wish Technology Company under specific pathogen-free conditions and a 12 h/12 h light/dark cycle. Five-week-old severe combined immunodeficient (SCID) female mice were used for the experiments. Mice were injected with exosomes (50 μg) collected from control, LSD1 KD, and LSD1 Rescue cells or control hnRNPA2B1 KD, and hnRNPA2B1 KD+miR-6881-3p mimics every 2 days for 3 weeks. Then, 5 × 10^6^ MCF7-Fluc cells (MCF7 cells stably expressing firefly luciferase) were suspended in 200 μL of phosphate-buffered saline (PBS) and injected into the mice *via* the caudal artery according to previous methods [33]. After 5 weeks, D-luciferin sodium salt (Yeasen, Shanghai, China) was intraperitoneal injected into the mice based on 150 mg/kg body weight. After 30 min, images were captured in vivo using the Tanon 5200 Automatic Chemiluminescence Imaging Analysis System (Tanon Science & Technology, Shanghai, China). The mice were subsequently sacrificed and the femur and tibia were extracted for ex vivo bioluminescence imaging. Luminescence quantification was performed using ImageJ software. The tibias of the mice were fixed and scanned with the high-resolution desktop micro-computed tomographer SKYSCAN 1276 (Bruker, Karlsruhe, Germany). Three-dimensional reconstruction of the scanned tibia was undertaken and the following parameters were calculated: bone mineral density (BMD), bone volume per tissue volume (BV/TV), trabecular separation (Tb.Sp), and trabecular number (Tb.N). Finally, the mouse tibias were transported to Wuhan Bioqiandu Technology Co., LTD (Wuhan, China) for decalcification, paraffin embedding, sectioning, hematoxylin-eosin (H&E) staining, tartrate-resistant acid phosphatase (TRAP) staining, and osteocalcin (OCN) immunohistochemistry.

### Immunofluorescence

MCF7 cells were seeded in 24-well plates on glass coverslips and allowed to reach 50% confluence. The cells were then washed three times with PBS, fixed in 4% neutral-buffered formalin for 10 min, and incubated with antibodies against TSG101 (Abcam, ab83, Cambridge, UK), hnRNPA2B1 (Abcam, ab283592), or KDM1/LSD1 (Abcam, ab31954) at 37 °C for 2 h. After washing three times with PBS, the cells were incubated with Alexa Fluor 555-conjugated goat anti-mouse IgG (H + L) cross-adsorbed secondary antibody (Invitrogen, A-21422), Alexa Fluor 488-conjugated goat anti-rabbit IgG (H + L) cross-adsorbed secondary antibody (Invitrogen, A-11008), Alexa Fluor 555-conjugated goat anti-rabbit IgG (H + L) cross-adsorbed secondary antibody (Invitrogen, A-21428), or Alexa Fluor 488-conjugated goat anti-mouse IgG (H + L) cross-adsorbed secondary antibody (Invitrogen, A-11001) at 37 °C for 1 h. Finally, the cells were washed three times with PBS and mounted on a slide with an anti-fluorescence quenching agent and DAPI.

### Small RNA sequencing

The cells were washed three times with PBS and lysed using TRIzol (Invitrogen). Small RNA sequencing was performed simultaneously (Shanghai Outdo Biotech Co., Ltd, Shanghai, China). Small RNA-seq data were deposited in the GEO database under the accession number GSE218200 and GSE212291.

### MiRNA extraction and quantitative real-time PCR (qPCR)

MiRNAs were obtained from MCF7 cells that had been seeded in 6-well plates using the EasyPure miRNA Kit (TransGen Biotech, Beijing, China), followed by reverse transcription undertaken with TransScript miRNA First-Strand cDNA Synthesis SuperMix (TransGen Biotech, Beijing, China). qPCR was performed with TransStart Green qPCR SuperMix (TransGen Biotech). U6 was used for normalization. The reverse primer used for real-time PCR came from TransScript miRNA First-Strand cDNA Synthesis SuperMix (Universal miRNA qPCR Primer); the forward primers are listed in Table S2.

### RNA extraction and qPCR

RAW264.7 cells or hBMSCs (8 × 10^5^) were seeded in a 6-well plate and, after attachment, 20 μg of exosomes was added to each well. After 48 h, the cells were washed three times with PBS. Total RNA was extracted from cells using TransZol Up (TransGen Biotech) and the RNA concentration was determined by Nanodrop (Allsheng Instruments Co., Ltd). Reverse transcription was performed using the EasyScript All-in-One First-Strand cDNA Synthesis SuperMix for qPCR (TransGen Biotech). qPCR was performed using TransStart Green qPCR SuperMix (TransGen Biotech). The sequences of the primers have been previously reported [22].

### MiRNA motif analysis

Improbizer (https://users.soe.ucsc.edu/~kent/improbizer/improbizer.html) was used for an unbiased search and analysis of six nucleotide-long motifs of miRNAs. A cladogram was generated with Web logo software (http://weblogo.threeplusone.com/).

### Alkaline phosphatase (ALP) staining and Alizarin Red S staining

HBMSCs were cultured with 10 μg/mL exosomes and were induced to differentiate into osteoblasts using TransDiffer Human Mesenchymal Stromal Cell Osteogenic Differentiation Medium (TransGen Biotech, Beijing, China). The medium was refreshed and exosomes were added every 5 days. After 7 days of culture, cells were fixed in 4% polyformaldehyde and stained using a BCIP/NBT Alkaline Phosphatase Color Development Kit (Beyotime Biotechnology, Shanghai, China). Meanwhile, osteoblast-lineage cells were also fixed after 14 days, and stained with Alizarin Red S (1%, PH 4.2, Solarbio, Beijing, China) at 37 °C for 1–2 h. Osteoblasts were imaged after washing with PBS to remove any remaining dye, images of osteoblasts. An equal volume of 2% hexadecyl pyridinium chloride monohydrate solution was added to lyse the cells, following which the absorbance was measured at 492 and 562 nm.

### ALP activity assays

HBMSCs were cultured with 10 μg/mL exosomes in osteoblastic differentiation medium for 5 days and the supernatant was collected. ALP activity in the supernatant was assessed using an ALP activity kit (Nanjing Jiancheng Bioengineering Institute, Nanjing, China).

### TRAP staining

RAW 264.7 cells were treated with 10 μg/mL exosomes and nuclear factor-kappa B ligand (RANKL) (Pepro Tech, New Jersey, NJ, USA) for 5 days. The cells were subsequently fixed in 4% paraformaldehyde for 10 min, washed three times with distilled water, and subjected to TRAP staining using a commercial kit (BestBio, Shanghai, China) according to the manufacturer’s protocol. TRAP+ mononuclear and multinucleated cells were identified as preosteoclasts and osteoclasts, respectively, and counted under an inverted microscope in five random visual fields.

### RNA immunoprecipitation (RIP) assay

The RIP assay was performed with a BersinBio RIP Kit (BersinBio, Guangzhou, China) following the manufacturer’s instructions. Briefly, Flag-tagged full-length and partial hnRNPA2B1 plasmids were synthesized by Comate Bioscience Co., Ltd. (Changchun, China). The cells were transfected with plasmids, lysed, and then incubated with an anti-Flag antibody (Sigma-Aldrich, F1804) overnight at 4 °C. Magnetic beads were then added to the lysate and, after 2 h of incubation, the RNA was purified and reverse transcribed using TransScript miRNA First-Strand cDNA Synthesis SuperMix (TransGen Biotech, Beijing, China). The levels of miR-6881-3p, miR-6726-3p, miR-34c-3p, and miR-4457 were analyzed using qPCR with IgG serving as a negative control.

### RNA pull-down

RNA pull-down was performed with a BersinBio miRNA Pulldown Kit (BersinBio, Guangzhou, China) according to the manufacturer’s instructions. Briefly, MCF7 cells were transfected with biotin-labeled miRNA probes (GenePharma Co., Ltd, Shanghai, China) and the EGFP-hnRNPA2B1 plasmid (Generay Biotechnology, Shanghai, China), lysed, and incubated with streptavidin magnetic beads. MiRNA-bound proteins were subsequently enriched by adsorption through a magnetic frame and the bound proteins were identified by western blot.

### Chromatin immunoprecipitation (ChIP)

ChIP was performed as described by Zhang et al. [34] using anti-LSD1 (Abcam, ab17721) and anti-H3K9me2 (Invitrogen, PA5-120810) antibodies. The PCR primers used in the ChIP analysis are listed in Table S3.

### Statistics

All data were expressed as means ± standard deviation (SD). Differences between two groups were assessed with the two-tailed Student’s unpaired t test. The one-way Anova with Tukey’s multiple comparison test was used to assess differences between more than two groups. No statistical methods were used to predetermine the sample size. Mice were randomly allocated to experimental groups. No blinding method was used for injection. There were no animal exclusion criteria. The variance was similar between the groups that were being statistically compared. Statistics were performed using Prism 8 (GraphPad, San Diego, USA) and significance is indicated in the figures.

### Supplementary information

Supplementary files

Original western blots

aj-checklist

## Data Availability

The data that support the findings of this study are openly available. Small RNA-seq data were deposited in the GEO database under the accession number GSE218200 and GSE212291.
