# Supplementary material for: LSD1 modulates the bone metastasis of breast cancer cells through hnRNPA2B1-mediated sorting of exosomal miRNAs
Source: Cell Death Discov. 2024 Mar 6;10:115. doi: 10.1038/s41420-024-01897-5 (PMC10917739; doi:10.1038/s41420-024-01897-5)
Supplement: Supplementary file 1 — Supplementary files [file 41420_2024_1897_MOESM1_ESM.docx]

**Supplementary Tables:**

**Table S1. siRNAs sequences (all sequences from 5’ to 3’)**

|  | Sense | Antisense |
| --- | --- | --- |
| sihnRNPA2B1 | GCUCUUUAUUGGUGGCUUATT | UAAGCCACCAAUAAAGAGCTT |
| siLSD1#1 | CUAUAAAGCUCCAAUACUGTT | CAGUAUUGGAGCUUUAUAGTT |
| siLSD1#2 | GCAAGGAAUAUGAUGAAUUTT | AATTCAUCAUAUUCCUUGCTT |

**Table S2.** **miRNAs qPCR primer sequences (all sequences from 5’ to 3’)**

|  | Forward | Reverse |
| --- | --- | --- |
| miR-6726-3p | CTCGCCCTGTCTCCCGCTAG | Universal miRNA qPCR Primer |
| miR-4457 | TCACAAGGTATTGACTGGCGTA | Universal miRNA qPCR Primer |
| miR-34c-3p | AATCACTAACCACACGGCCAGG | Universal miRNA qPCR Primer |
| miR-6881-3p | ATCCTCTTTCGTCCTTCCCACT | Universal miRNA qPCR Primer |

**Table S3.** **ChIP primer sequences (all sequences from 5’ to 3’)**

|  | Forward | Reverse |
| --- | --- | --- |
| hnRNPA2B1-Pro 1 | ACTGGGGATCCTGGTACCTAAA | TTGGGTAATCGTGCCTGGTT |
| hnRNPA2B1-Pro 2 | GTCATTGCGGCGTGAACAAT | TCCCTGCAGTTACGCCAATG |
| hnRNPA2B1-Pro 3 | GGTTCTAGAAAAGCGGCGG | GCCGAGCGAGATGAGAGAGAT |
| hnRNPA2B1-3’UTR | GGTCTTTGTAAGAGTGTAGAAGCA | TCTTAACTCTACACACGCACTT |

**Supplementary Figures and legends:**

**
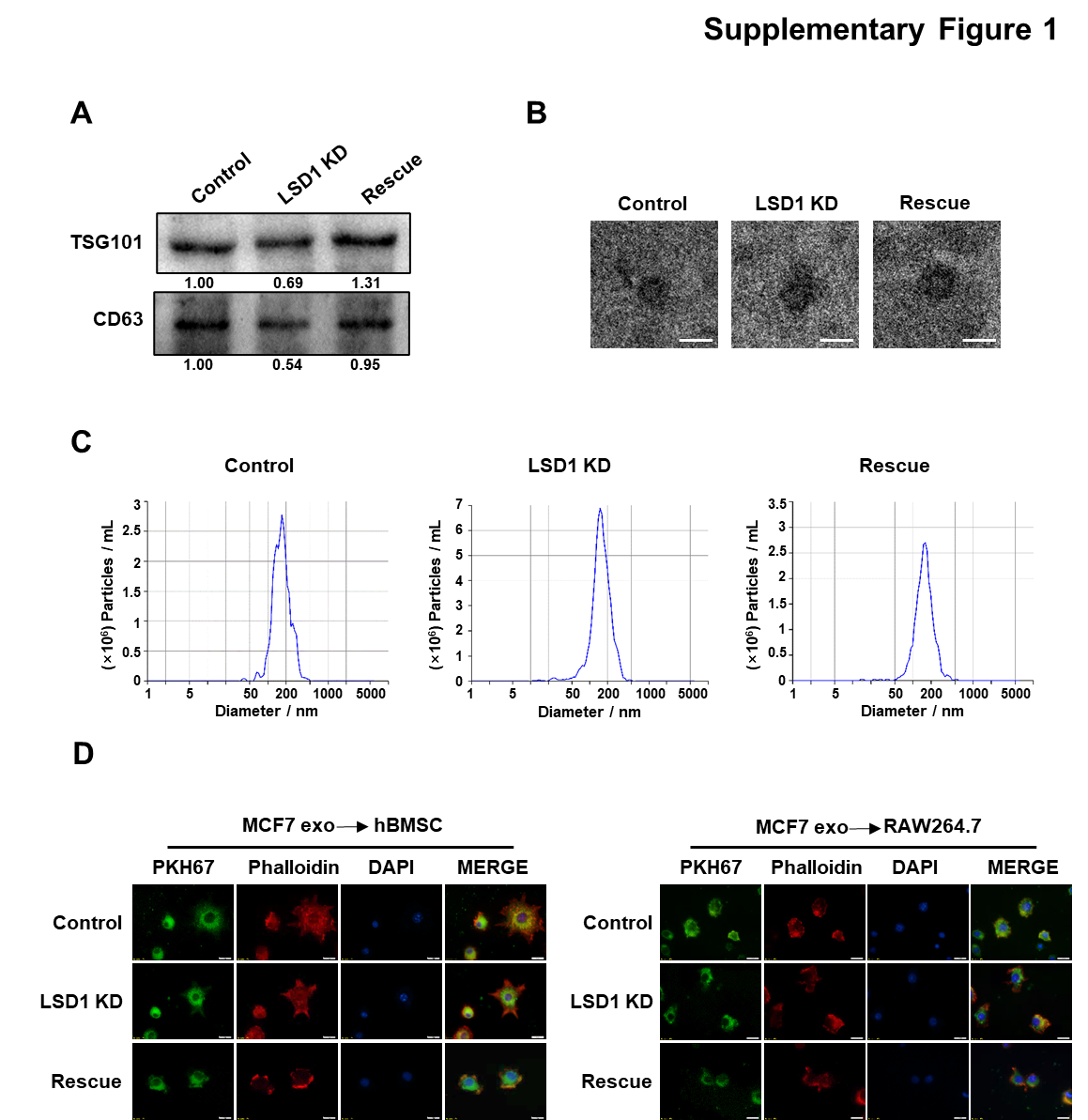
**

**Fig. S1. Identification of exosomes from breast cancer cells.**

(A) Western blots showing expression of exosome markers CD63 and TSG101 in the purified exosomes from MCF7 cells without (control) or with LSD1 knockdown (LSD1 KD), and LSD1 KD cells with restored LSD1 expression (rescue). The number below the band represents the value from densitometry reading, relative to the control, which was set at 1.00. (B) Morphology of exosomes from control, LSD1 KD and rescue cells under electron microscopy (scale bars: 100 nm). (C) Nanoparticle Tracking analysis (NTA) of the diameter and concentration of exosomes from control, LSD1 KD and rescue cells. Immunofluorescence images of PKH67 labeled exosomes that are absorbed by hBMSC cells and RAW264.7 cells (D). (green: PKH67; red: Phalloidin; blue: DAPI; scale bars: 10 μm)

**
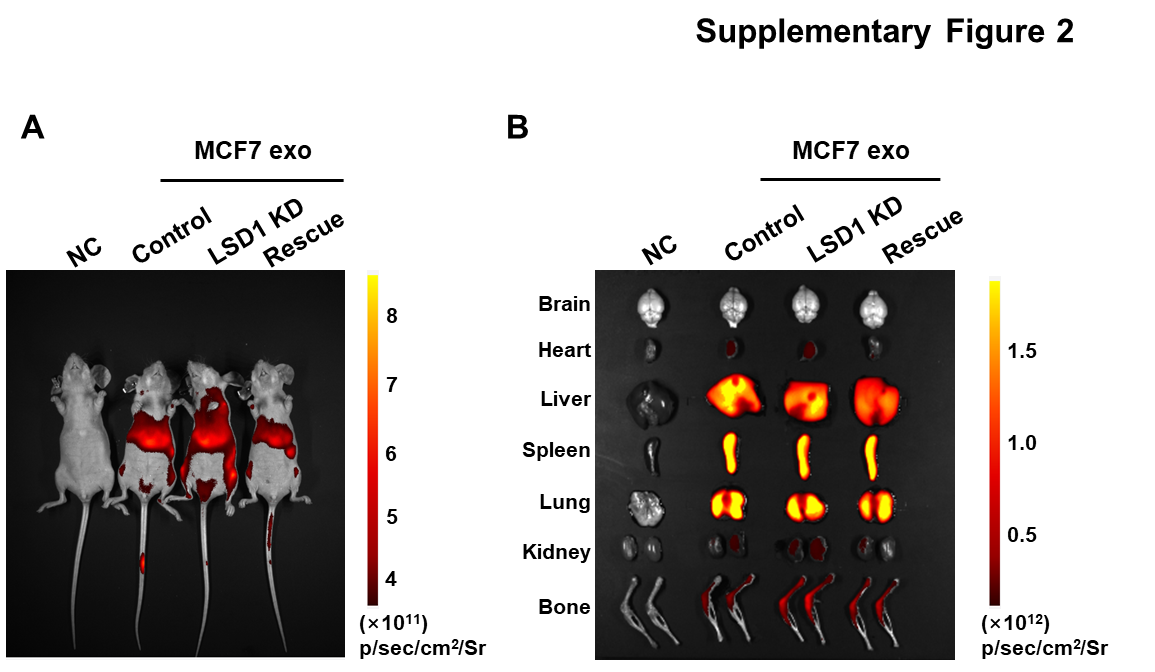
**

**Fig. S2. Exosomes from control, LSD1 KD, and rescue breast cancer cells** **exhibited the propensity to reach the bone.** (A) The fluorescence signal distribution *in vivo* with PBS or DiR-labeled exosomes derived from indicated cells. (B) The fluorescence signal distribution in multiple organs from mice injected with PBS or DiR-labeled exosomes derived from indicated cells.

**
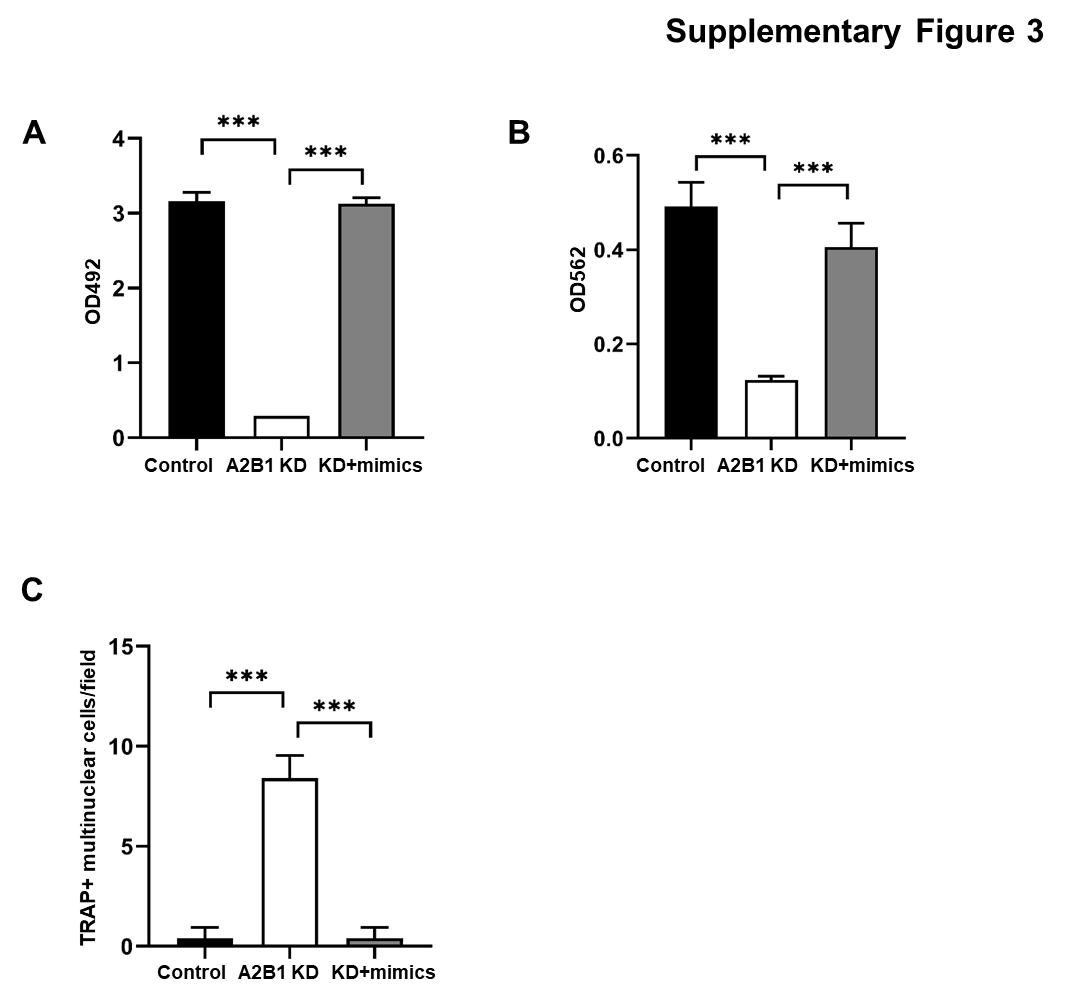
**

**Fig. S3. MiR-6881-3p rescued the effects of hnRNPA2B1 knockdown (KD) exosomes on osteoblast and osteoclast differentiation.** (A) The absorbance of dissolved Alizarin Red S staining at 492 nm in the control, hnRNPA2B1 KD, and hnRNPA2B1 KD+miR-6881-3p mimics groups. (B) The absorbance of dissolved ALP staining at 562 nm in the control, hnRNPA2B1 KD, and hnRNPA2B1 KD+miR-6881-3p mimics groups. (C) Quantification of TRAP+ cells in the control, hnRNPA2B1 KD, and hnRNPA2B1 KD+miR-6881-3p mimics groups. *** indicates *P* < 0.001.


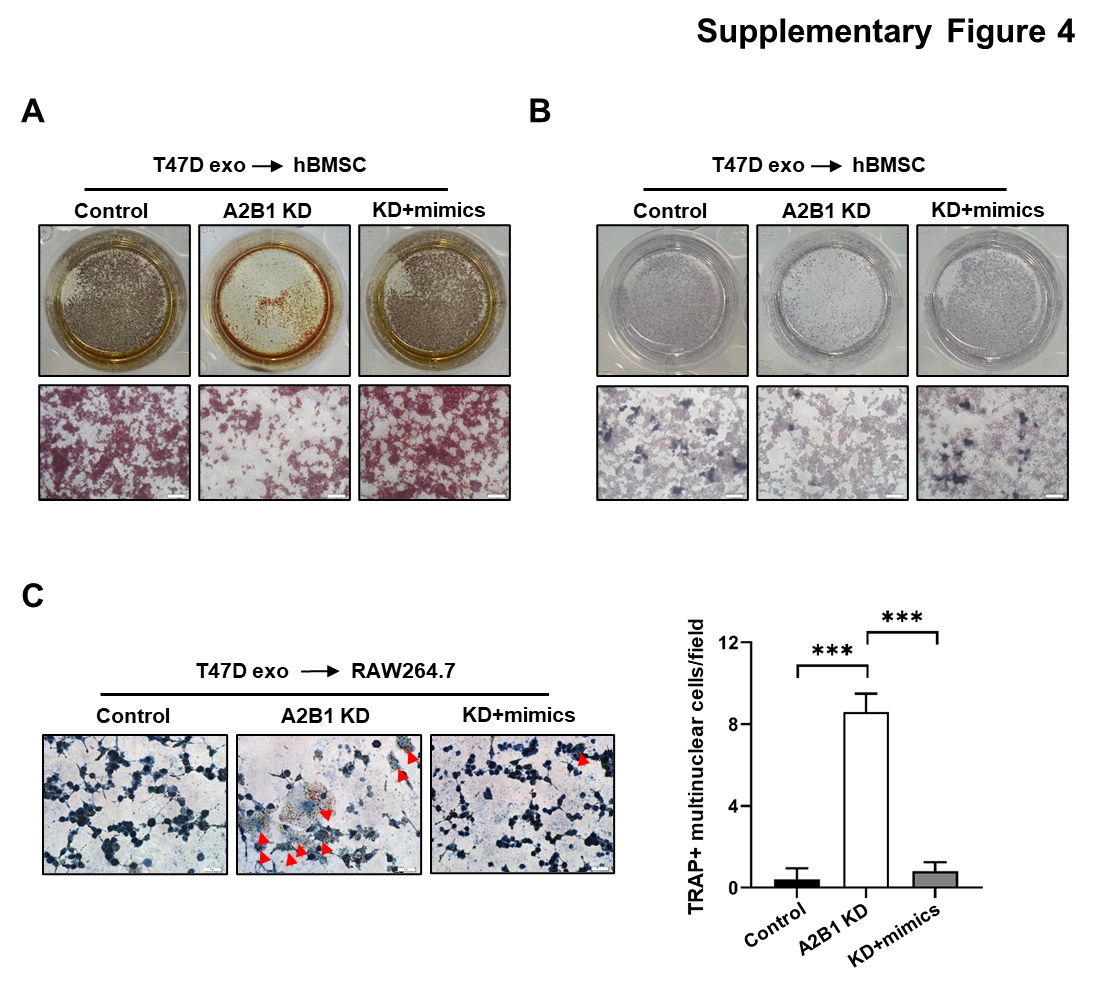


**Fig. S4. HnRNPA2B1 KD inhibited osteoblast differentiation and promoted osteoclast differentiation by downregulating miR-6881-3p in exosomes from T47D cells.** Representative images of Alizarin Red staining (A) and ALP staining (B) (scale bars: 50 μm). (C) TRAP staining in RAW264.7 cells treated with exosomes derived from control, hnRNPA2B1 KD, or hnRNPA2B1 KD+miR-6881-3p mimics-treated induced to osteoclast differentiation (scale bars: 20 µm).


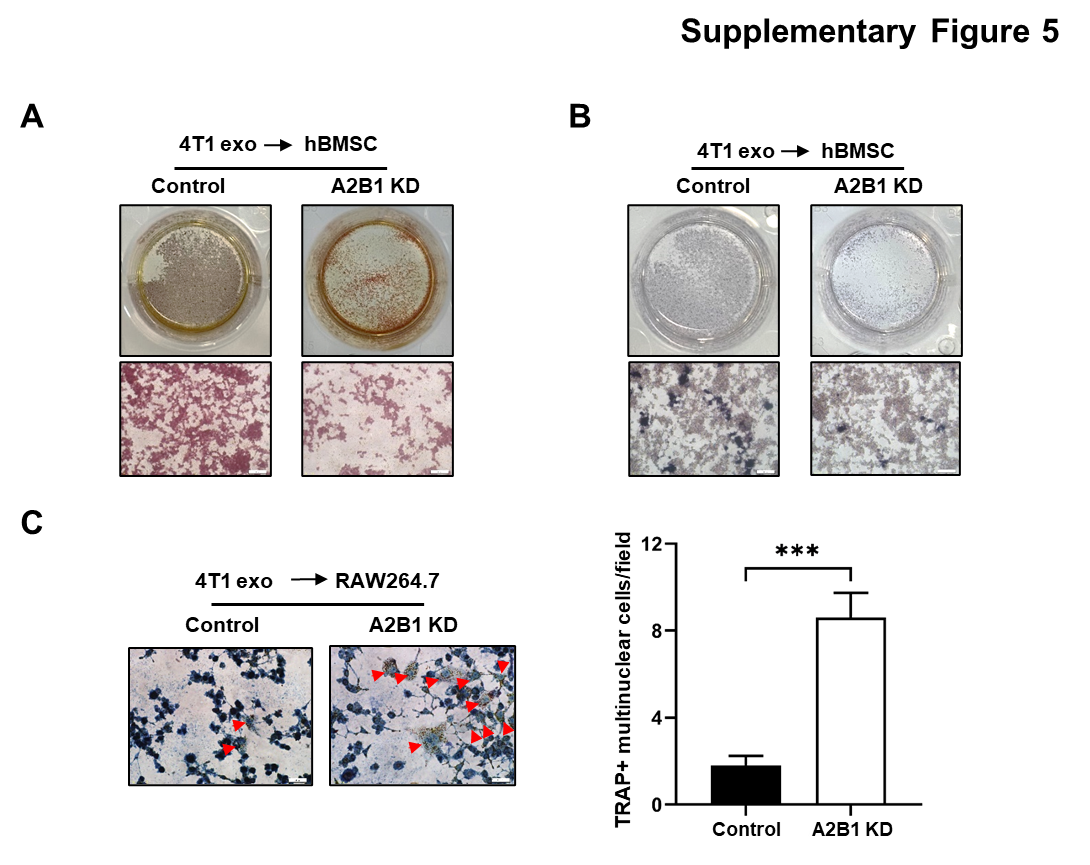


**Fig. S5. HnRNPA2B1 KD exosomes inhibited osteoblast differentiation and promoted osteoclast differentiation from 4T1 cells.** Representative images of Alizarin Red staining (A) and ALP staining (B) (scale bars: 50 μm). (C) TRAP staining in RAW264.7 cells treated with exosomes derived from control or hnRNPA2B1 KD induced to osteoclast differentiation (scale bars: 20 µm).
