# Supplementary figures and images for: LSD1 modulates the bone metastasis of breast cancer cells through hnRNPA2B1-mediated sorting of exosomal miRNAs
Source: Cell Death Discov. 2024 Mar 6;10:115. doi: 10.1038/s41420-024-01897-5 (PMC10917739; doi:10.1038/s41420-024-01897-5)

Fig 4 B

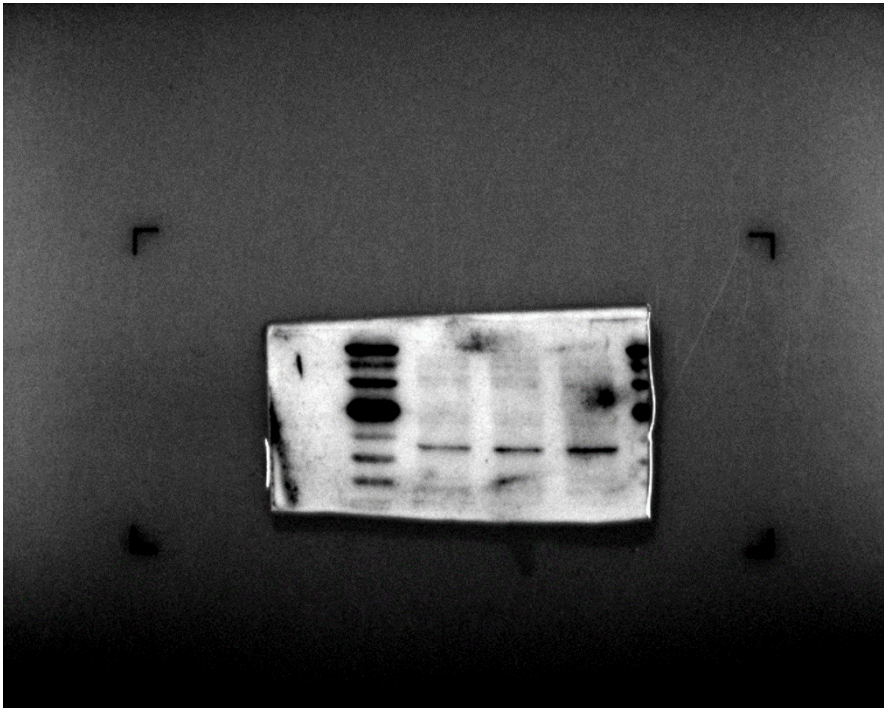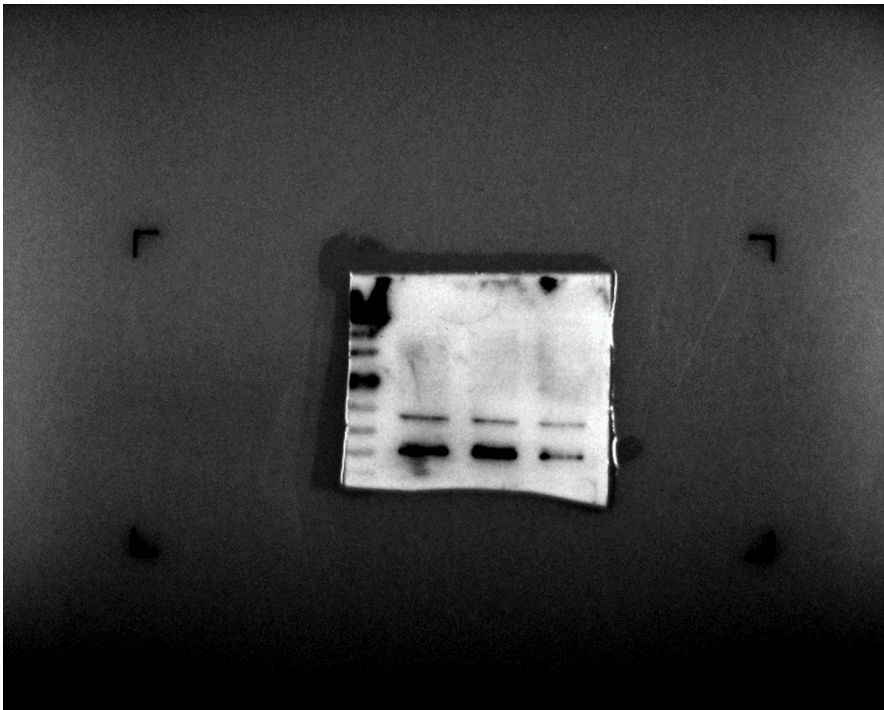

Fig 4 G

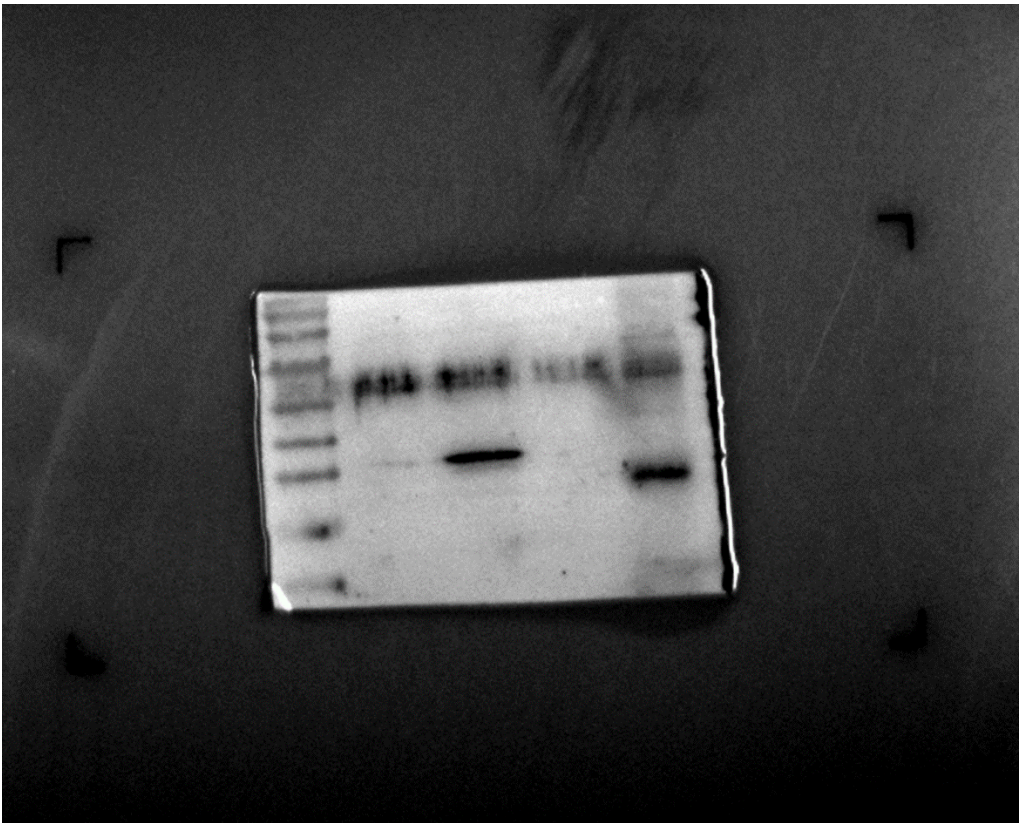

Fig 5 A

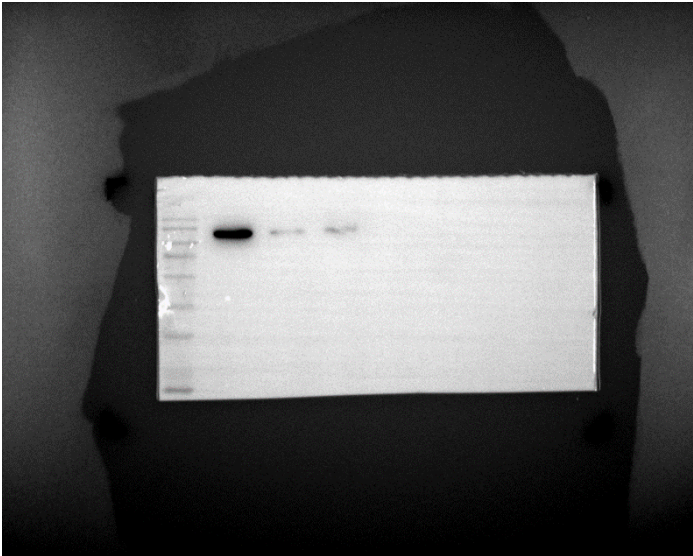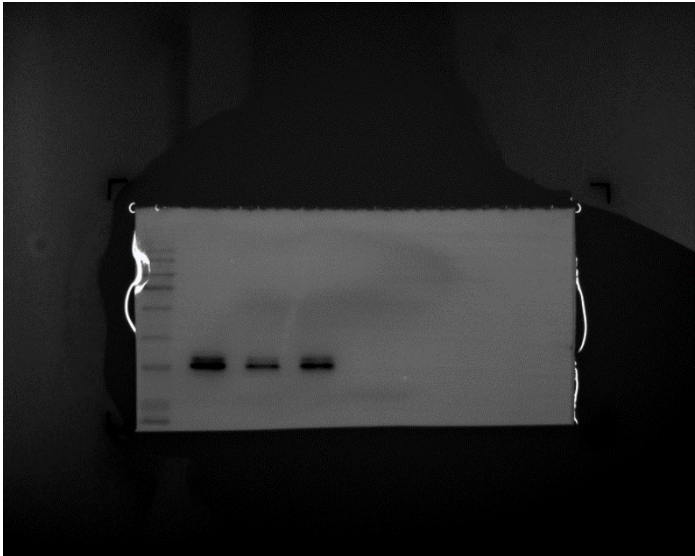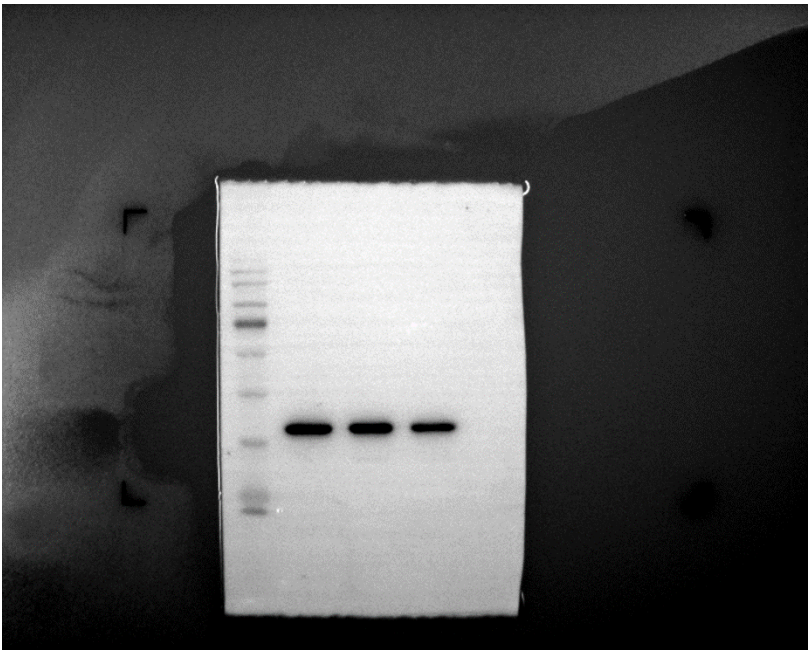

Supplementary Fig 1 A

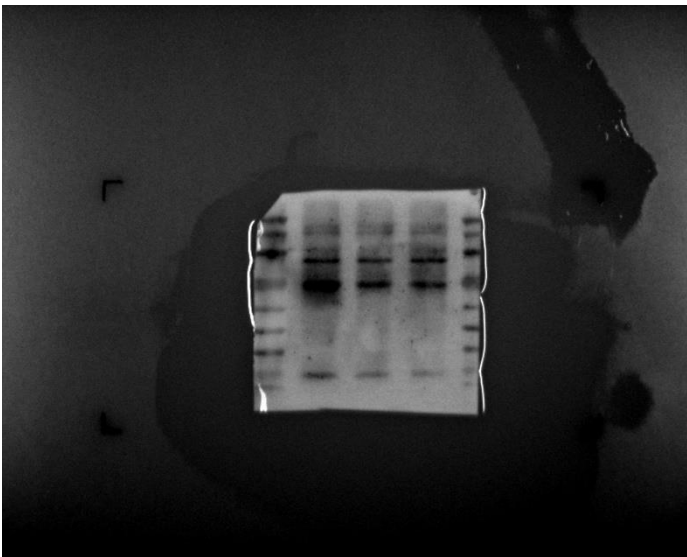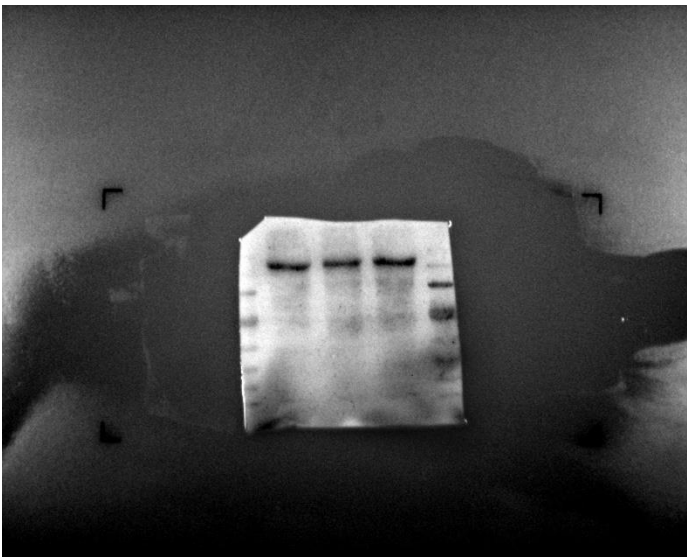

Supplement: Supplementary file 2 — Original western blots [file 41420_2024_1897_MOESM2_ESM.pdf]
